# Vision Impairment Among Late Mid-Life Women: Michigan Study of Women’s Health Across the Nation

**DOI:** 10.1093/geroni/igaa057.2951

**Published:** 2020-12-16

**Authors:** Carrie Karvonen-Gutierrez, Navasuja Kumar, Sayoko Moroi, David Musch, Sarah Dougherty Wood, Joshua Ehrlich, Michelle Hood

**Affiliations:** 1 University of Michigan, Ann Arbor, Michigan, United States; 2 The Ohio State University College of Medicine, Columbus, Ohio, United States; 3 University of Michigan School of Public Health, Ann Arbor, Michigan, United States

## Abstract

There are few data on mid-life sensory health and its contribution to health and disability outcomes. The Study of Women’s Health Across the Nation—Michigan site (N=252 women, mean age=66.0 years) collected comprehensive data regarding ocular health to estimate the burden and correlates of vision impairment (VI) and unmet need for vision correction in this population. The prevalence of VI based on presenting and best-corrected visual acuity was 11.1% (95% CI 7.5,15.7) and 2.8% (95% CI 1.1,5.6), respectively. Thus, 75.0% of presenting VI was correctable, yet only 29.0% of women wore an appropriate eyeglass prescription. Black women and those with greater financial strain or less education were more likely to have presenting VI, but there were no differences in best-corrected VI. This presentation will include a discussion of the contributions of SWAN to the study of sensory aging, as well as methodological considerations for research from a mid-life aging cohort. Part of a symposium sponsored by Sensory Health Interest Group.

